# Supplementary material for: Comparative Genomics and Phylogenetic Analysis of the Chloroplast Genomes in Three Medicinal Salvia Species for Bioexploration
Source: Int J Mol Sci. 2022 Oct 11;23(20):12080. doi: 10.3390/ijms232012080 (PMC9603726; doi:10.3390/ijms232012080)
Supplement: Supplementary file 1 [file ijms-23-12080-s001.zip › Figure S3. Structure comparison of the 23Salvia cpgenomes20221008.pdf]

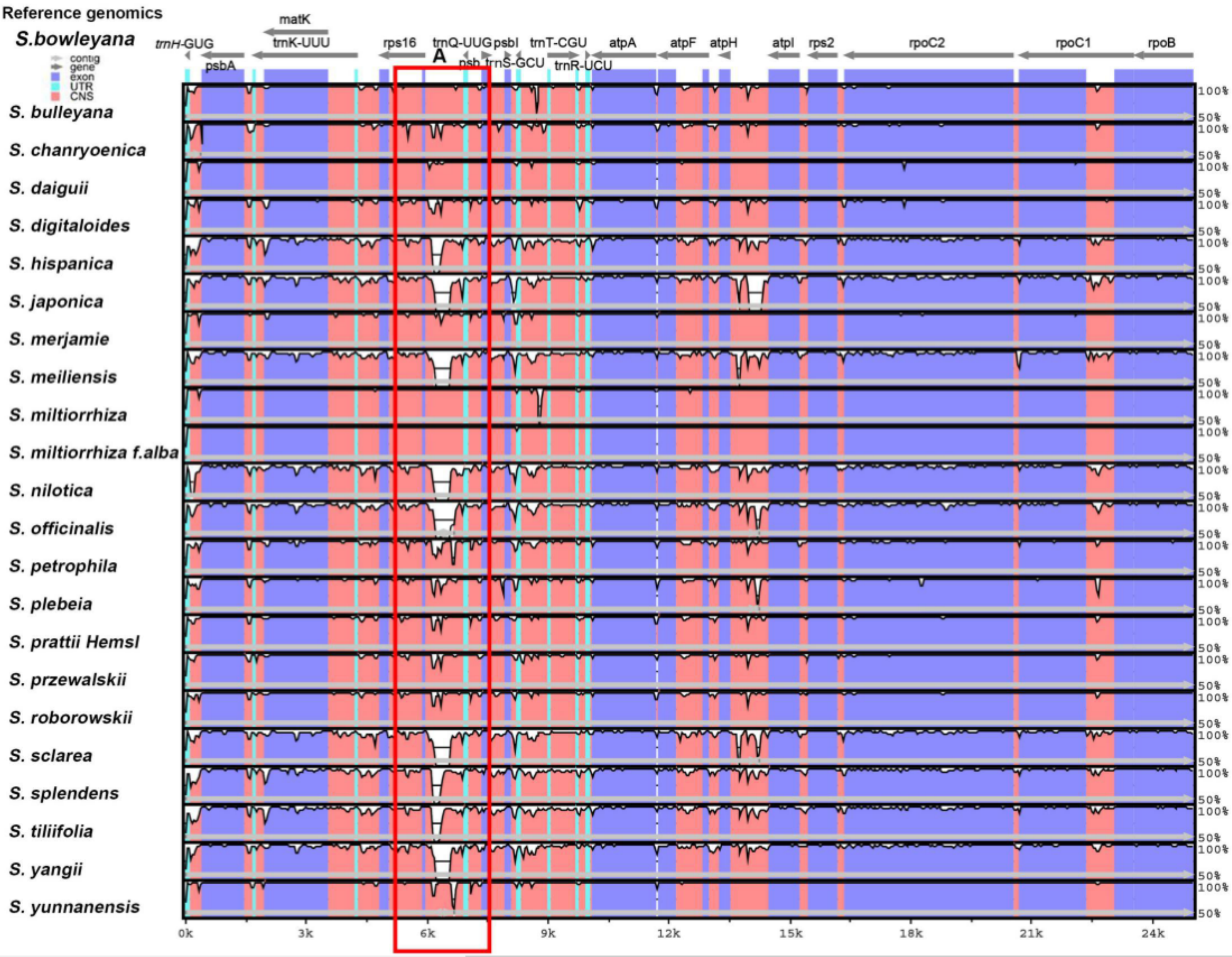

Reference genomics
S.bowleyana
contig
gene
exon
UTR
CNS
matK
trnH-GUG
psbA
trnK-UUU
rps16
trnQ-UUG
psbI
trnT-CGU
atpA
atpF
atpH
atpI
rps2
rpoC2
rpoC1
rpoB
A
trnS-GCU
trnR-UCU
S. bulleyana
S. chanryoenica
S. daiguii
S. digitaloides
S. hispanica
S. japonica
S. merjamie
S. meiliensis
S. miltiorrhiza
S. miltiorrhiza f.alba
S. nilotica
S. officinalis
S. petrophila
S. plebeia
S. prattii Hemsl
S. przewalskii
S. roborowskii
S. sclarea
S. splendens
S. tiliifolia
S. yangii
S. yunnanensis
100%
50%
0k
3k
6k
9k
12k
15k
18k
21k
24k

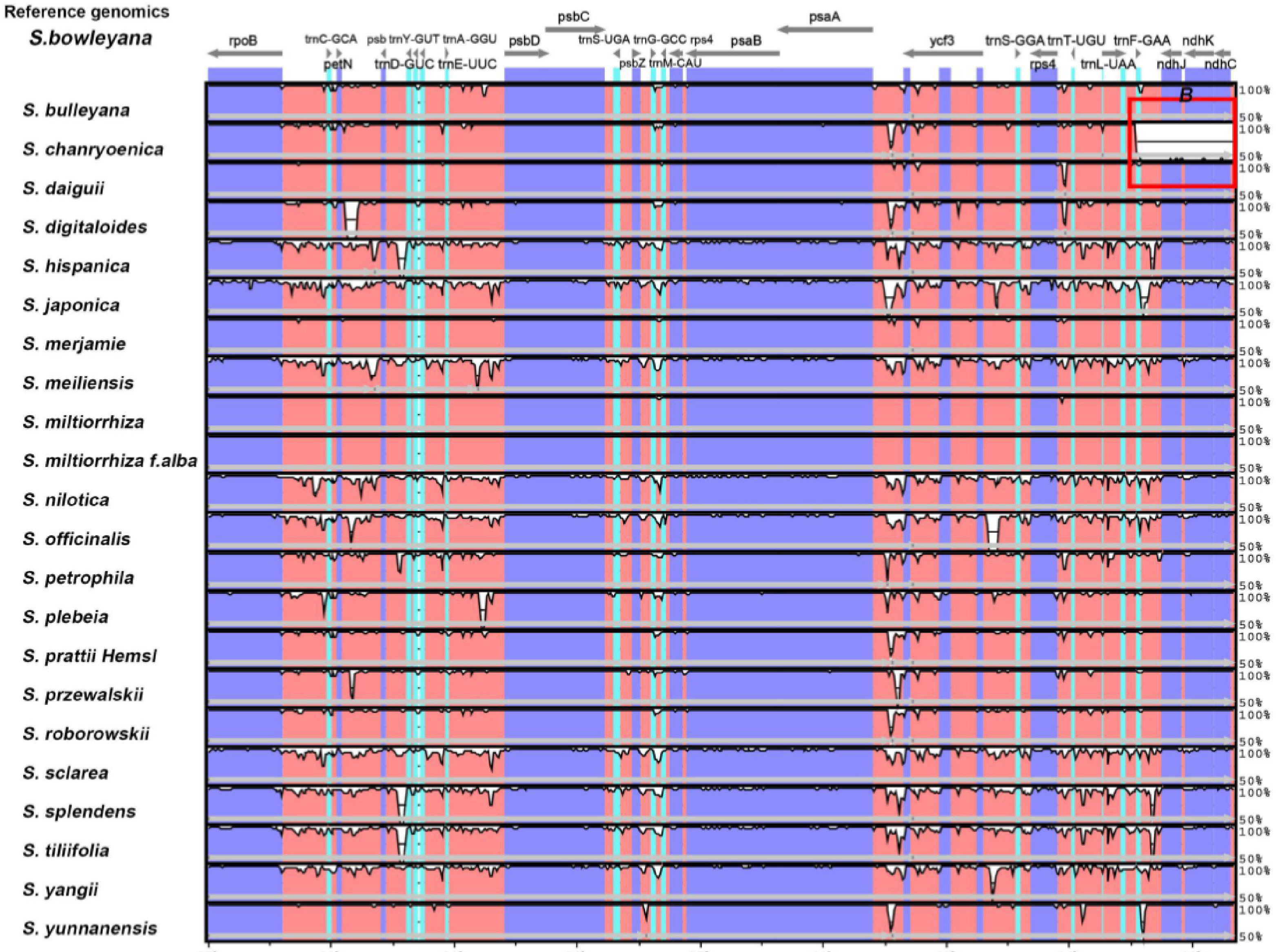

Reference genomics
S.bowleyana
rpoB
trnC-GCA
petN
psb
trnY-GUT
trnD-GUC
trnA-GGU
trnE-UUC
psbD
psbC
trnS-UGA
psbZ
trnG-GCC
trnM-CAU
rps4
psaB
psaA
ycf3
trnS-GGA
rps4
trnT-UGU
trnL-UAA
trnF-GAA
ndhJ
ndhK
ndhC
B
S. bulleyana
S. chanryoenica
S. daiguii
S. digitaloides
S. hispanica
S. japonica
S. merjamie
S. meiliensis
S. miltiorrhiza
S. miltiorrhiza f.alba
S. nilotica
S. officinalis
S. petrophila
S. plebeia
S. prattii Hemsl
S. przewalskii
S. roborowskii
S. sclarea
S. splendens
S. tiliifolia
S. yangii
S. yunnanensis
100%
50%
25k
28k
31k
34k
37k
40k
43k
46k
49k

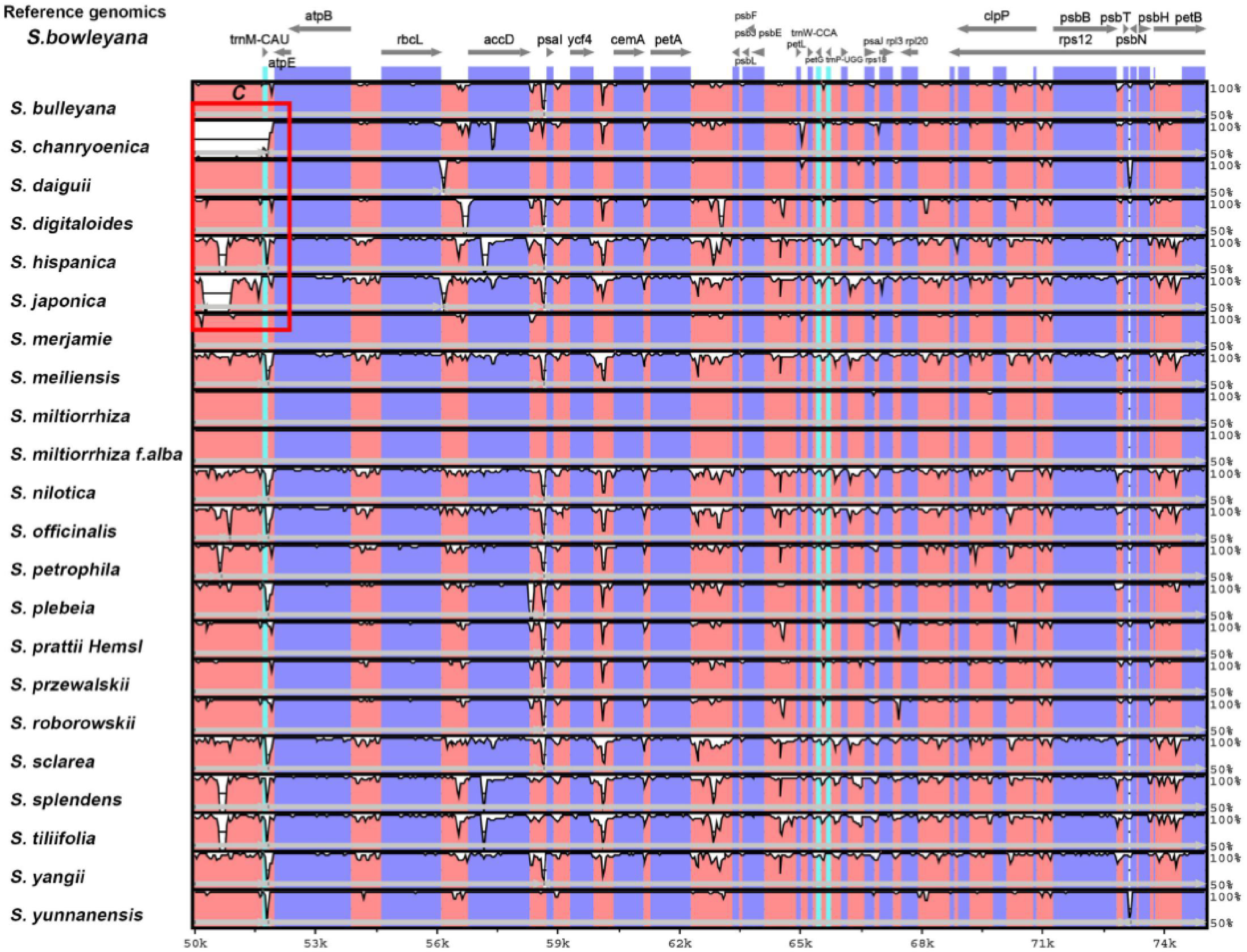

Reference genomics
S.bowleyana
trnM-CAU
atpE
atpB
rbcL
accD
psaI
ycf4
cemA
petA
psbF
psbJ
psbE
psbL
petL
trnW-CCA
petG
trnP-UGG
psaJ
rpl33
rps18
rpl20
clpP
psbB
rps12
psbT
psbN
psbH
petB
C
S. bulleyana
S. chanryoenica
S. daiguii
S. digitaloides
S. hispanica
S. japonica
S. merjamie
S. meiliensis
S. miltiorrhiza
S. miltiorrhiza f.alba
S. nilotica
S. officinalis
S. petrophila
S. plebeia
S. prattii Hemsl
S. przewalskii
S. roborowskii
S. sclarea
S. splendens
S. tiliifolia
S. yangii
S. yunnanensis
100%
50%
50k
53k
56k
59k
62k
65k
68k
71k
74k

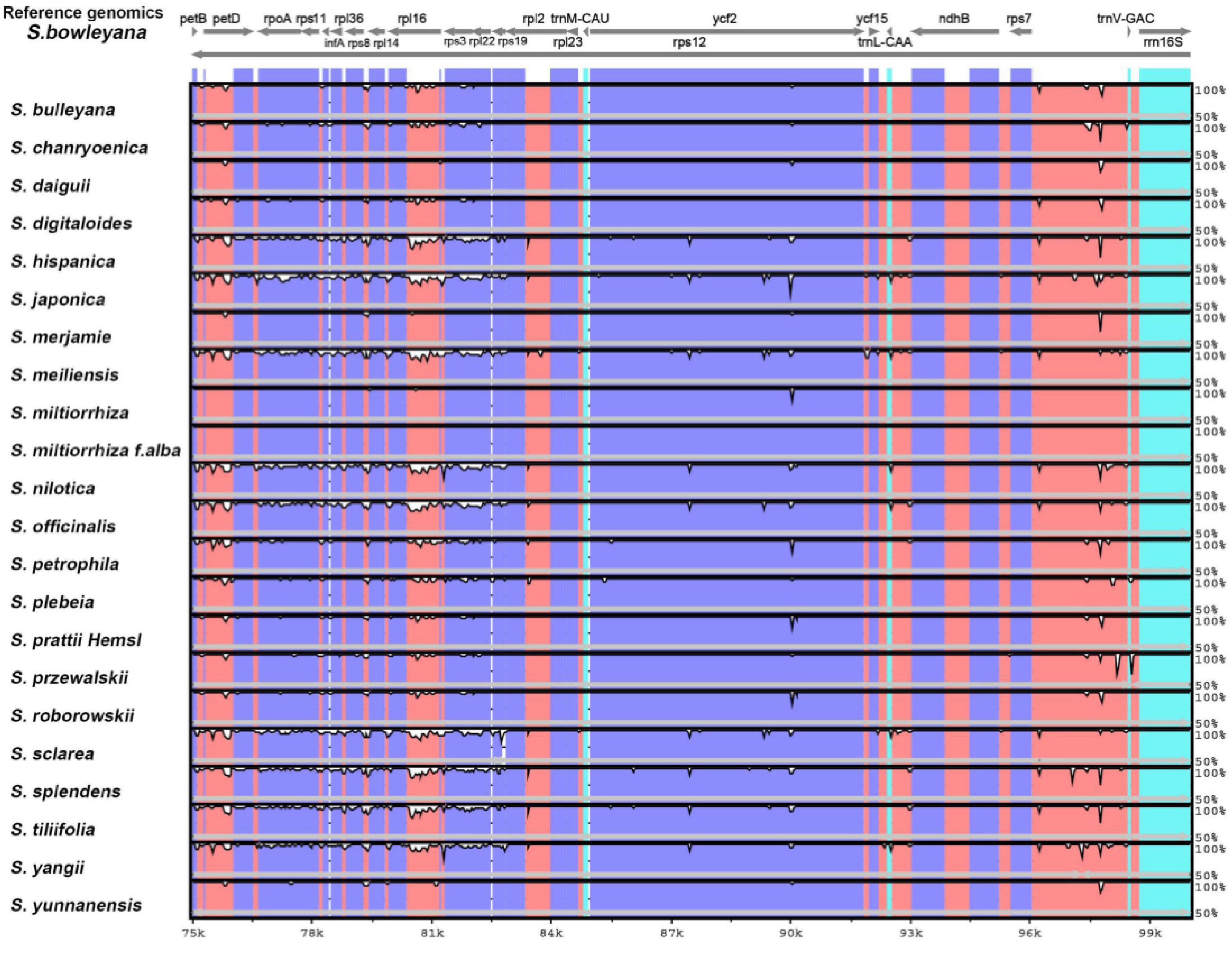

Reference genomics
S.bowleyana
petB
petD
rpoA
rps11
rpl36
rpl16
rpl2
trnM-CAU
ycf2
ycf15
ndhB
rps7
trnV-GAC
infA
rps8
rpl14
rps3
rpl22
rps19
rpl23
rps12
trnL-CAA
rrn16S
S. bulleyana
S. chanryoenica
S. daiguii
S. digitaloides
S. hispanica
S. japonica
S. merjamie
S. meiliensis
S. miltiorrhiza
S. miltiorrhiza f.alba
S. nilotica
S. officinalis
S. petrophila
S. plebeia
S. prattii Hemsl
S. przewalskii
S. roborowskii
S. sclarea
S. splendens
S. tiliifolia
S. yangii
S. yunnanensis
100%
50%
75k
78k
81k
84k
87k
90k
93k
96k
99k

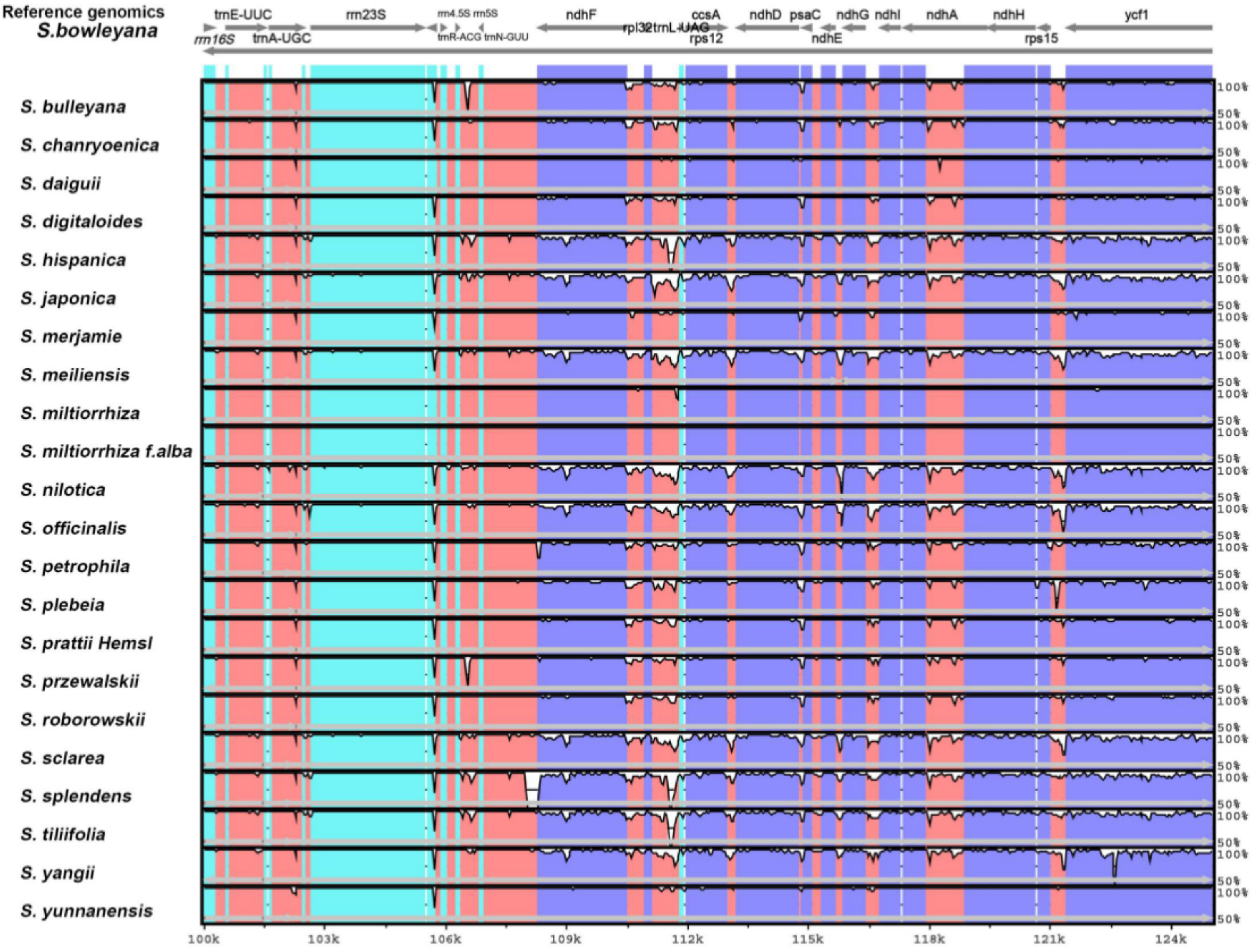
Reference genomics
S.bowleyana
trnE-UUC
rrn23S
rrn4.5S rrn5S
ndhF
ccsA
ndhD
psaC
ndhG
ndhI
ndhA
ndhH
ycf1
rrn16S
trnA-UGC
trnR-ACG trnN-GUU
rpl32trnL-UAG
rps12
ndhE
rps15
S. bulleyana
S. chanryoenica
S. daiguii
S. digitaloides
S. hispanica
S. japonica
S. merjamie
S. meiliensis
S. miltiorrhiza
S. miltiorrhiza f.alba
S. nilotica
S. officinalis
S. petrophila
S. plebeia
S. prattii Hemsl
S. przewalskii
S. roborowskii
S. sclarea
S. splendens
S. tiliifolia
S. yangii
S. yunnanensis
100%
50%
100k
103k
106k
109k
112k
115k
118k
121k
124k

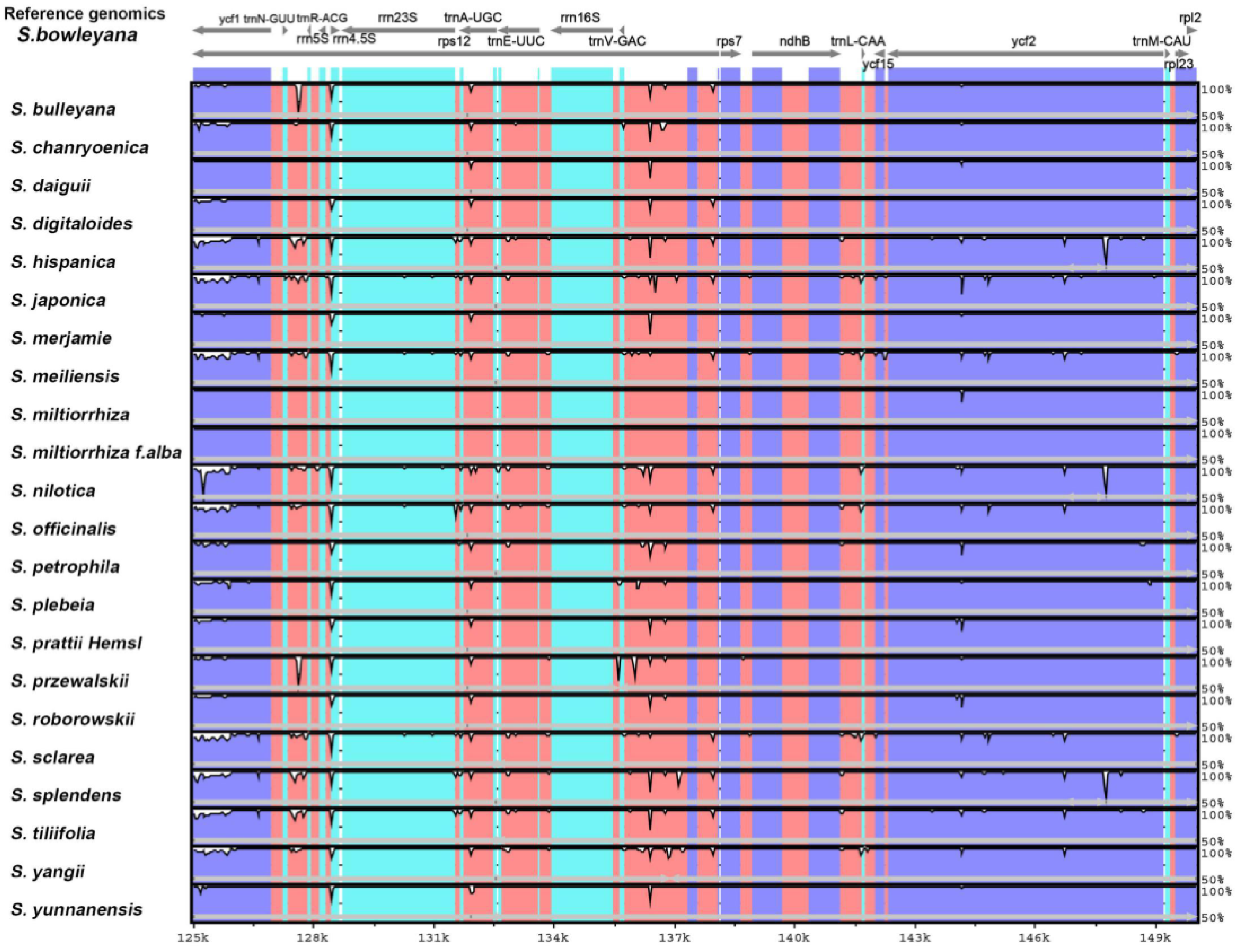

Figure S3. Structure comparison of the 23Salvia cpgenomes20221008
