# Supplementary figures and images for: Comparative Genomics and Phylogenetic Analysis of the Chloroplast Genomes in Three Medicinal Salvia Species for Bioexploration
Source: Int J Mol Sci. 2022 Oct 11;23(20):12080. doi: 10.3390/ijms232012080 (PMC9603726; doi:10.3390/ijms232012080)

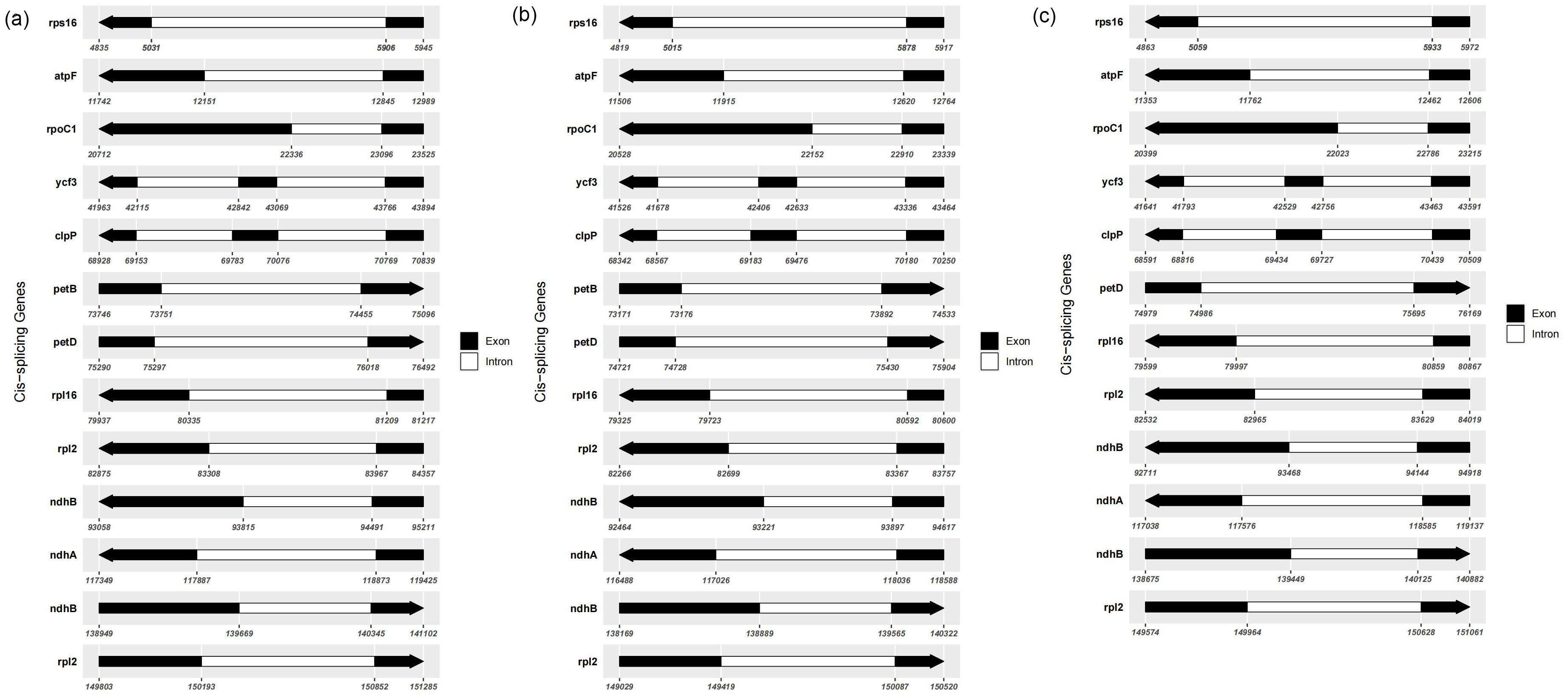

Supplement: Supplementary file 1 [file ijms-23-12080-s001.zip › Figure S1. Schematic presentation of cis-splicing genes (CDS) structure in the S. bowleyana, S. splendens, S. officinalis cpgenome.jpg]

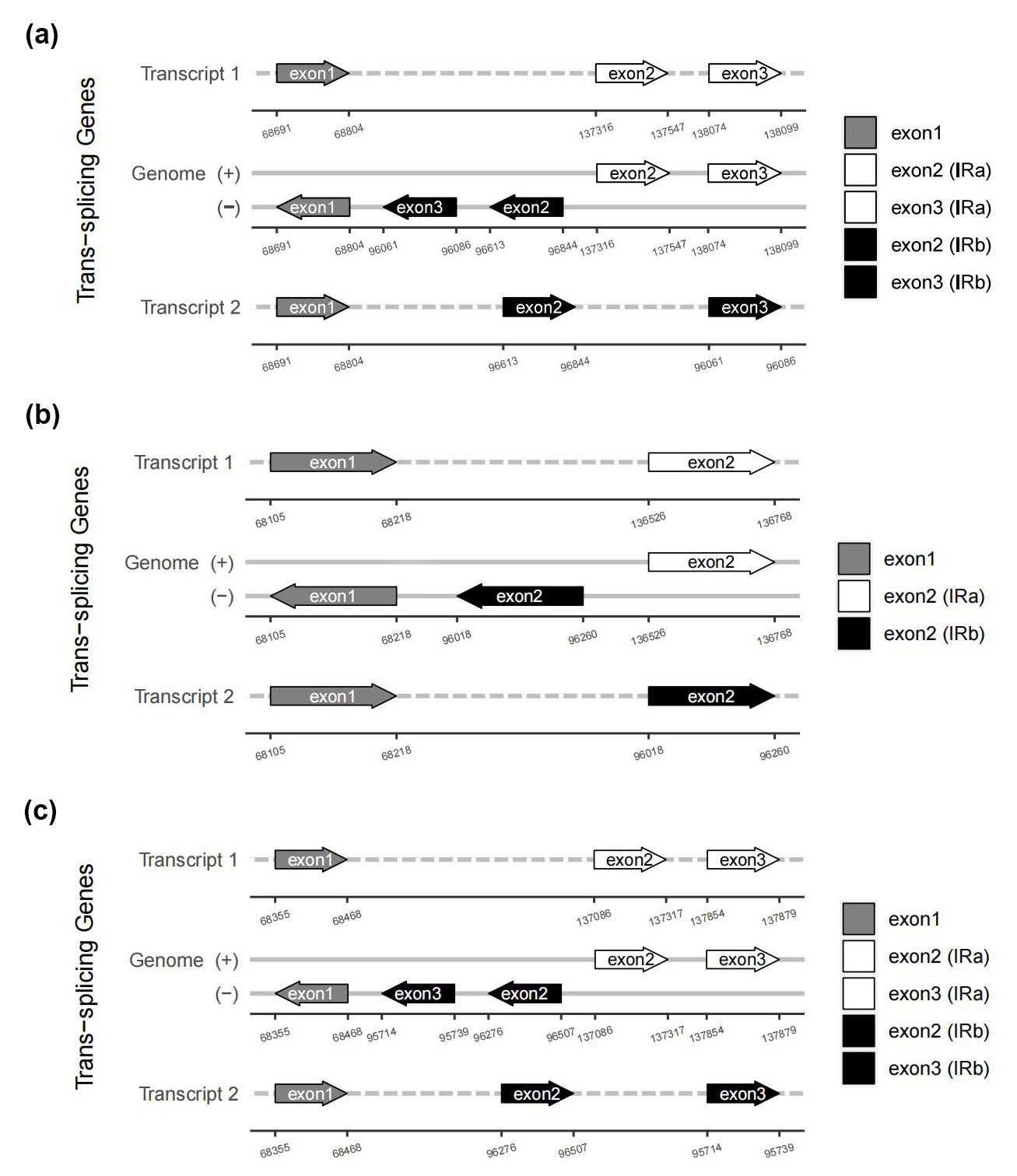

Supplement: Supplementary file 1 [file ijms-23-12080-s001.zip › Figure S2. Schematic presentation of the trans-splicing genes in the S. bowleyana, S. splendens, S. officinalis cpgenome.jpg]

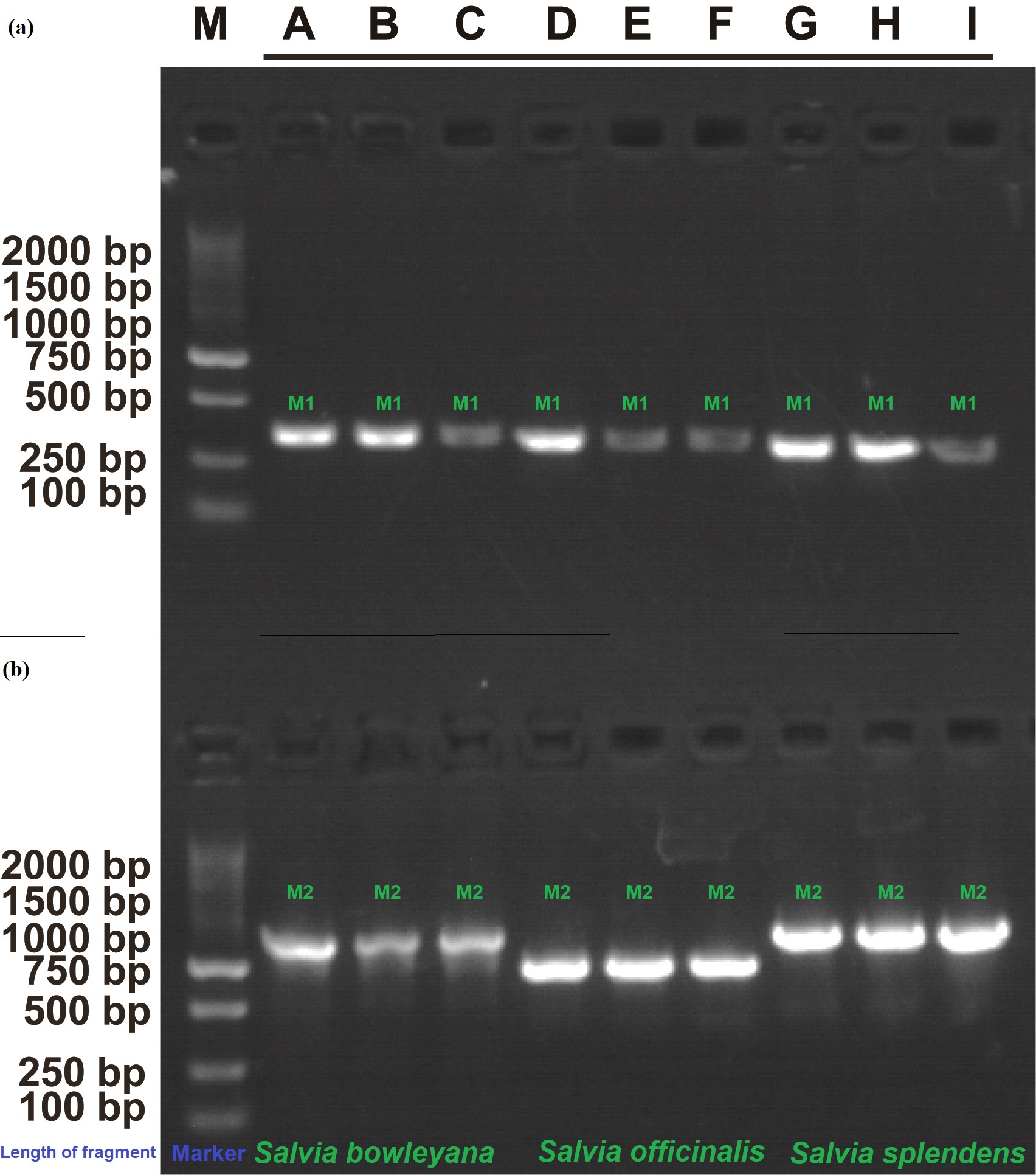

Supplement: Supplementary file 1 [file ijms-23-12080-s001.zip › Figure S4. The cloning amlification products of the two IGS in the three studied Salvia species.jpg]
